# Supplementary material for: Adjusting the neuron to astrocyte ratio with cytostatics in hippocampal cell cultures from postnatal rats: A comparison of cytarabino furanoside (AraC) and 5-fluoro-2’-deoxyuridine (FUdR)
Source: PLoS One. 2022 Mar 9;17(3):e0265084. doi: 10.1371/journal.pone.0265084 (PMC8906639; doi:10.1371/journal.pone.0265084)
Supplement: S2 File — (ZIP) [file pone.0265084.s002.zip › S2_Description of Automated Analysis.pdf]

S2 Description of image analysis using CellProfiler and CellProfiler Analyst

General Information about the Data Acquisition and Analysis Procedure

Samples were prepared and acquired as described in the methods section. At first, images were analyzed unblinded and manually. The results of this original manual analysis are depicted figures 1-3.

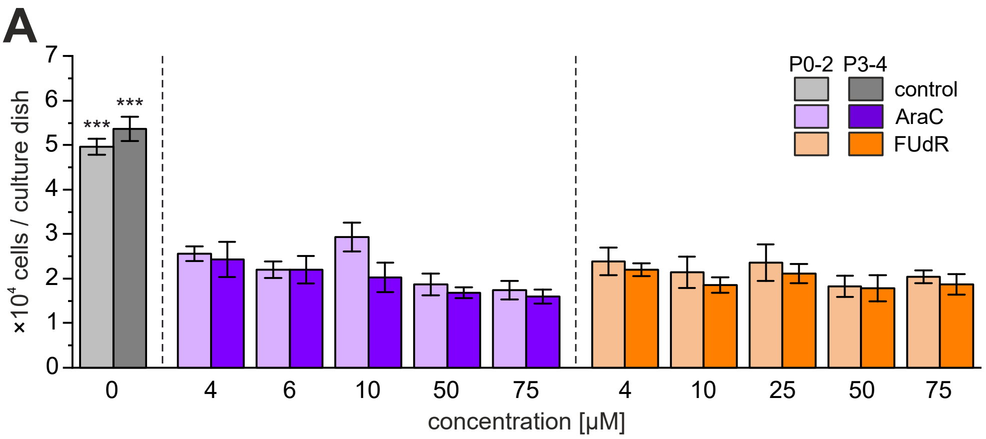

Figure 1: Total cell numbers in cultures obtained from early postnatal (P0-2) and older (P3-4) animals. Bars represent means ± SEM. Statistical significances of the decreases in cell numbers with respect to control cultures were $p < 0.001$ (***) for all concentrations.

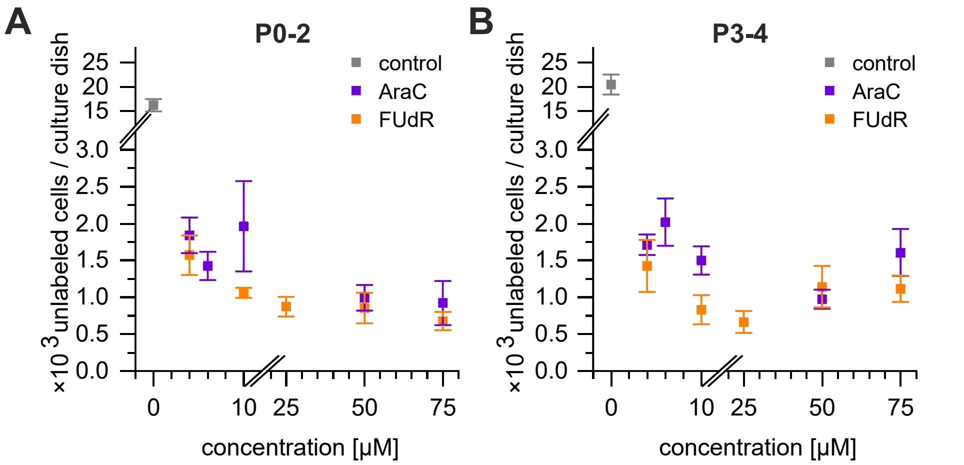

Figure 2: Number of unlabeled GFAP-negative and β3-tubulin-negative cells in P0-2 (A) and P3-4 (B) cultures. This population was significantly reduced in all cytostatic-treated cultures ($p < 0.001$) with respect to control cultures (not indicated by asterisks to enhance clarity).

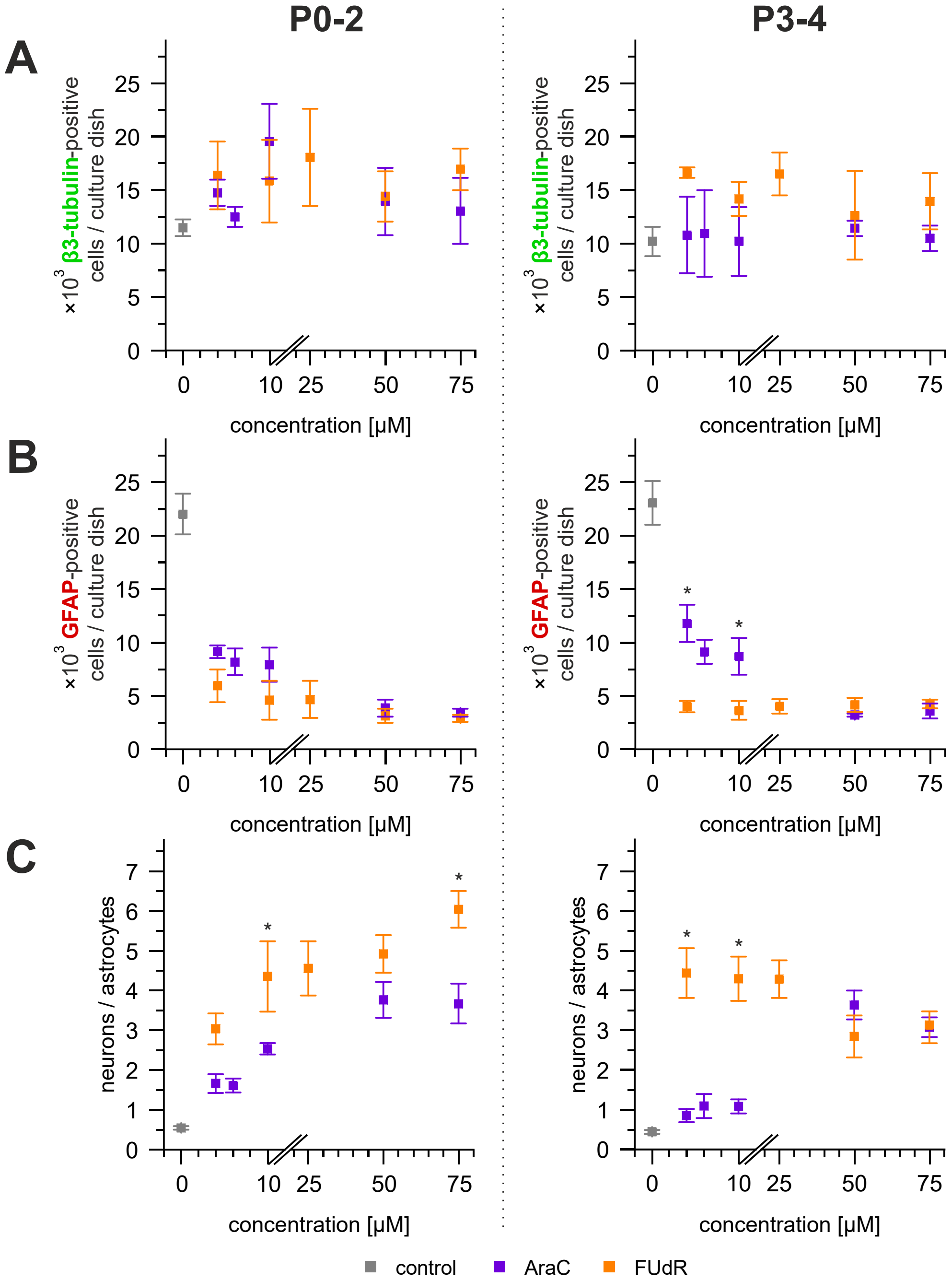

*Figure 3: (A) Number of beta-III-tubulin-positive cells, and (B) the number of GFAP-positive cells. (C) Ratio of β3-tubulin-positive cells (neurons) to GFAP-positive cells (astrocytes). Left column shows data obtained from P0-2 cultures, right column data from P3-4 cultures. Data points represent mean ± SEM of at least three independent preparations. Each preparation consisted of three independent cultures per condition. Statistically significant differences between FUdR and AraC-treated cultures are marked with * ($p < 0.05$). Decreases in GFAP-positive astrocytes with respect to control cultures ($p < 0.001$) and of the increases in the neuron to astrocyte ratio ($p < 0.05$) were consistently significantly smaller for all cytostatic concentrations tested (not shown for clarity).*

However, to avoid the introduction of personal bias, images were analyzed again using the open-source programs CellProfiler [1-4] and CellProfiler Analyst [5-8].

Figures shown in the body text of the publication are based on the results acquired with CellProfiler (Analyst) and exclude the treatment conditions 6 µM AraC and 25 µM FudR. These two treatment conditions were excluded as experiments with these treatment concentrations were only executed with one of the cytostatica each instead of both. Furthermore, an exclusion of these treatment conditions did not alter the overall conclusions drawn from the data. However, the results from the automated analysis of the cell cultures treated according to these conditions are still included in the uploaded result table ('ResultTable_ExperimentalImages').

The automated image analysis was performed as follows:

To ensure that pipelines and classifier model created with CellProfiler and CellProfiler Analyst were unbiased, the scientist (AH) creating and applying them to the data received the experimental images with unsorted and encoded filenames. These file names (‚four-digit-number_letter' e.g. ‚1000_P') could not be connected to an acquisition date or cell culture treatment. The only information extractable from the image files was the type of microscope used for acquisition as one microscope setup acquired color and the other monochrome images. The number in the file names was used to identify images of the same cell culture section and the letter to identify the type of image (fluorescence or phase contrast) and staining (Hoechst, tubulin or GFAP): During image acquisition each randomly chosen and observed cell culture section was imaged four times to acquire a phase contrast image (P), an image capturing the nuclei stained with Hoechst 33258 (H), an image capturing the beta-III-tubulin staining (T) and an image capturing the GFAP staining (G). To further ensure an unbiased analysis, the CellProfiler pipelines and CellProfiler Analyst model were created using a training set of data, which were created separately from the described experiments. This training set contained images acquired on the same microscopes as the

experiment images and of cell cultures, which were prepared in the same manner as the cultures of the experiment.

After analysis with CellProfiler and CellProfiler Analyst, image data was connected again to the respective cell culture treatments by the scientist (HL) who originally encoded the file names. He then proceeded to interpret the data.

CellProfiler (4.2.1) Pipelines

Two pipelines (‚FDUR_AraC_convertIX51tograyscale.cppipe‘ and ‚FDUR_AraC.cppipe‘) were created for image analysis with CellProfiler. Both pipelines used the ‚Metadata‘ module to extract the section number (first four digits of file name) and image/staining type (last letter of file name) from the file name. The ‚NamesAndTypes‘ module was applied to assign a more descriptive name to images according to the image/staining type (e.g. ‚phase_contrast‘ instead of ‚P‘). The ‚Groups‘ module was used to group the four images of the same section as identified by the ‚Metadata‘ module.

The first pipeline (‚FDUR_AraC_convertIX51tograyscale.cppipe‘) was used to convert the color (RGB) images into grayscale images (module: ColorToGray) and save the converted images (module: SaveImages).

The second pipeline (‚FDUR_AraC.cppipe‘) was used for image segmentation and data extraction. The module ‚IdentifyPrimaryObjects‘ was applied to segment the Hoechst 33258 images (‚H‘ or ‚hoechst‘) and thereby identify cell nuclei, which would later on be used as objects for classification in CellProfiler Analyst. The module was set to only consider identified objects with a diameter range of 20 to 80 pixel units and to discard objects touching the border of the image. These settings were applied to exclude objects, which were either too small or big to represent a single cell nucleus, and to avoid falsifying the later on extracted image data (‚Measure…‘ modules) by including cut off nuclei (objects touching the image border). Furthermore, the advanced settings of the ‚IdentifyPrimaryObjects‘ module were used to optimize the segmentation algorithm. A two-

class, adaptive Otsu algorithm was chosen for thresholding. The exact settings are documented in the uploaded pipeline. Even after careful adjustment of the available settings, there were still a few misidentified nuclei. Four types of segmentation errors occurred: some clumped cells could not be distinguished and were identified as a single cell (‚error_clumps'), larger cell debris was misidentified as a nuclei (‚error_debris'), a few oddly shaped or very large nuclei were identified as multiple nuclei instead of one (‚error_too_many') and due to the quality of some images (light reflections, blurriness, shadows) some nuclei were identified in parts of the images, where there were none (‚error_no_cell'). Later on in the analysis, CellProfiler Analyst was used to recognize and exclude these segmentation errors from the data set.

The modules ‚MeasureGranularity', ‚MeasureObjectIntensity', ‚MeasureObjectSizeShape', ‚MeasureTexture', and ‚MeasureColocalization' were applied to extract data from all four images of one section connected to the previously identified nuclei.

Finally, the ‚ExportToDatabase' module was used to save the extracted data into a SQLite database file and create a CellProfiler Analyst properties file.

CellProfiler Analyst (3.0.4) Classification Model

The ‚Classifier' tool of CellProfiler Analyst was used to manually classify 9680 identified nuclei from the training set into the categories ‚b3tubulin' (neurons; 2160 nuclei), ‚gfap' (astrocytes; 2238 nuclei), ‚unstained' (neither neurons nor astrocytes; 2304 nuclei) and the four above described segmentation errors (error_clumps: 755, error_debris: 658, error_too_many: 880, error_no_cell: 685). This classified training set was used to train a RandomForest Classifier algorithm. The trained algorithm model was then evaluated and the results displayed in a confusion matrix (figure 4). The overall classification accuracy was determined to be 89.89 %.

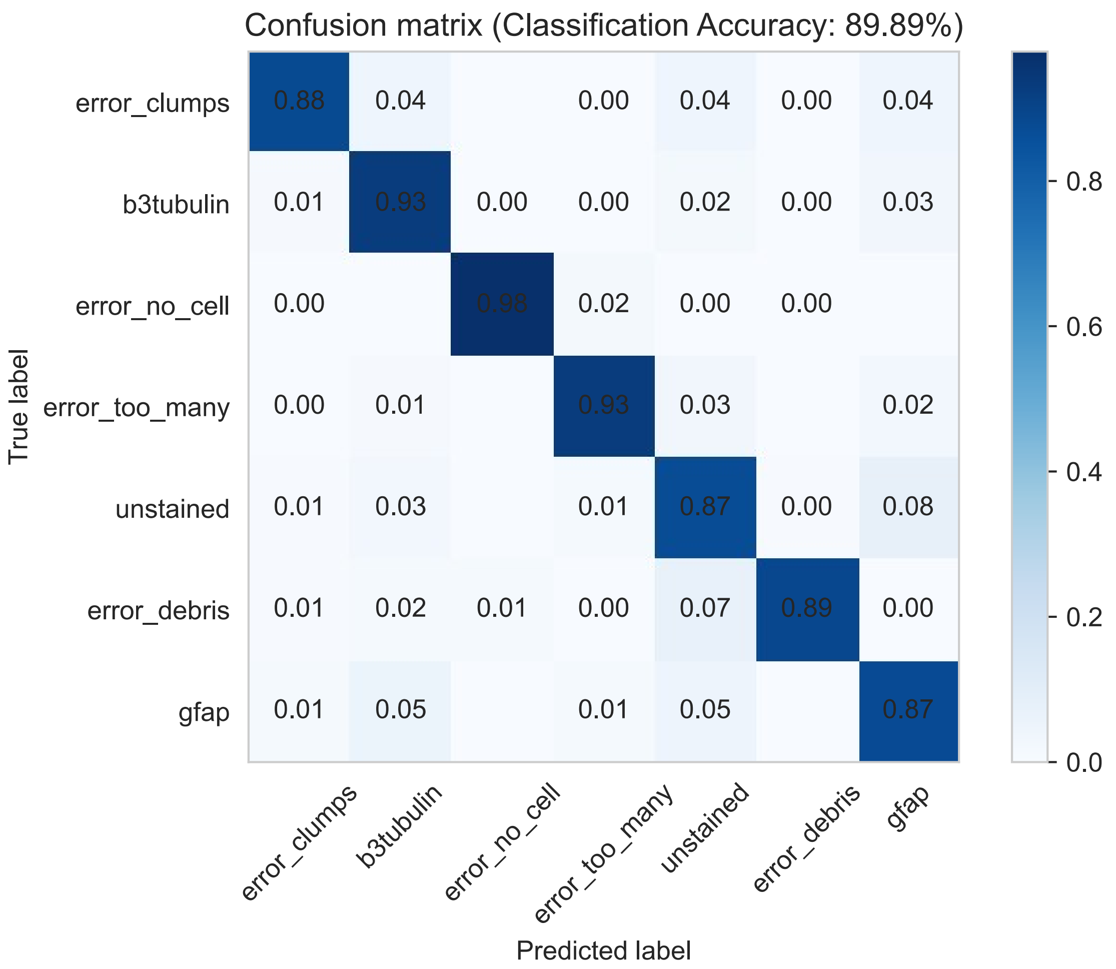

Figure 4: Confusion matrix created with CellProfiler Analyst to evaluate the accuracy of the trained RandomForest Classifier. The overall classification accuracy was determined to 89.89 % with accuracies for single labels varying from 87 to 98 %.

To identify the cells in the experimental images, the created classifier model was applied to the experimental image set. Result tables of the classification of the training set ('ResultTable_TrainingSet') and experiment images ('ResultTable_ExperimentalImages') are uploaded to the supplementary material.

Nuclei identified as one of the four segmentation errors were subtracted from the number of counted nuclei to yield the total cell number, while the number of nuclei classified as ‚b3tubulin', ‚gfap', and ‚unstained' yielded the number of neurons, astrocytes and neither neurons nor astrocytes.

Further Data Processing

As the amount of analyzed images was too large (2580 imaged cell culture sections) to manually check every single classified nucleus, the results created with CellProfiler Analyst were visually validated for random images on a test basis and some sections were excluded from the experiment due to a larger amount of misclassified cells (over about 30 %). A total of 30 sections (equaling 1.2 % of the original experimental image set) were excluded from the data set. These excluded sections are marked in grey in the uploaded table ('ResultTable_ExperimentalImages').

References

1. Stirling DR, Swain-Bowden MJ, Lucas AM, Carpenter AE, Cimini BA et al. (2021) CellProfiler 4: improvements in speed, utility and usability. BMC bioinformatics 22 (1): 433.

2. McQuin C, Goodman A, Chernyshev V, Kamentsky L, Cimini BA et al. (2018) CellProfiler 3.0: Next-generation image processing for biology. PLoS biology 16 (7): e2005970.

3. Kamentsky L, Jones TR, Fraser A, Bray M-A, Logan DJ et al. (2011) Improved structure, function and compatibility for CellProfiler: modular high-throughput image analysis software. Bioinformatics (Oxford, England) 27 (8): 1179–1180.

4. Carpenter AE, Jones TR, Lamprecht MR, Clarke C, Kang IH et al. (2006) CellProfiler: image analysis software for identifying and quantifying cell phenotypes. Genome biology 7 (10): R100.

5. Stirling DR, Carpenter AE, Cimini BA (2021) CellProfiler Analyst 3.0: Accessible data exploration and machine learning for image analysis. Bioinformatics (Oxford, England).

6. Jones TR, Carpenter AE, Lamprecht MR, Moffat J, Silver SJ et al. (2009) Scoring diverse cellular morphologies in image-based screens with iterative feedback and machine learning. Proceedings of the National Academy of Sciences of the United States of America 106 (6): 1826–1831.

7. Dao D, Fraser AN, Hung J, Ljosa V, Singh S et al. (2016) CellProfiler Analyst: interactive data exploration, analysis and classification of large biological image sets. Bioinformatics (Oxford, England) 32 (20): 3210–3212.

8. Jones TR, Kang IH, Wheeler DB, Lindquist RA, Papallo A et al. (2008) CellProfiler Analyst: data exploration and analysis software for complex image-based screens. BMC bioinformatics 9: 482.
